# Factors associated with food label use among Saudi adults: the roles of nutritional knowledge, educational level, and dietary practices

**DOI:** 10.3389/fnut.2026.1967330

**Published:** 2026-10-29

**Authors:** Amwaj Abugamza, Noura Aldous, Dana Alsahli, Kholoud Sahhari, Lujain Alshahrani, Areej Al-Hussain, Nahla Mohammed Bawazeer

**Affiliations:** Department of Health Sciences, College of Health and Rehabilitation Sciences, Princess Nourah bint Abdulrahman University, Riyadh, Saudi Arabia

**Keywords:** dietary practices, education, food label, nutritional knowledge, Saudi Arabia

## Abstract

**Background:**

Food labels are an important public health tool for promoting healthier food choices and reducing the burden of diet-related chronic diseases. However, the factors influencing food label use are not fully understood in Saudi Arabia.

**Aim:**

To investigate the factors associated with food label use among Saudi adults.

**Methods:**

A cross-sectional study was conducted among Saudi adults aged 18 years or older in Saudi Arabia. Participants were recruited using non-probability convenience sampling, supplemented by snowball sampling through social media. A translated and expert-reviewed questionnaire with acceptable internal consistency was used to collect data on sociodemographic characteristics, objective and subjective nutritional knowledge, food label use, and dietary practices. Food label use was assessed using a nine-item scale covering label reading, information checked, and the influence of labels on food choices. Each item was scored from 1 to 5, yielding a total score ranging from 9 to 45, with higher scores indicating greater food label use. Multivariable linear regression was performed to identify factors independently associated with food label use. A sensitivity analysis was conducted by replacing objective nutritional knowledge with subjective nutritional knowledge to examine associations with food label use across both measures.

**Results:**

A total of 683 participants were included in the final analysis (mean age: 30.8 ± 11.9 years). Higher objective nutritional knowledge (*B* = 0.715, 95% CI: 0.220–1.209, *p* = 0.004), having a diploma or bachelor’s degree compared with a high school education or below (*B* = 1.677, 95% CI: 0.182–3.172, *p* = 0.028) were associated with higher food label use scores. Not following a specific diet was associated with lower food label use scores (*B* = −2.324, 95% CI: −2.866 to −1.783, *p* < 0.001). These associations remained consistent in the sensitivity analysis using subjective nutritional knowledge.

**Conclusion:**

Nutritional knowledge, educational level, and dietary practices were associated with food label use among Saudi adults. Integrating nutrition literacy interventions with existing food labeling policies may enhance consumers’ engagement with food labels and support healthier food choices.

## 1 Introduction

Food labels are widely recognized as an important public health strategy for helping consumers make informed food choices and encouraging healthier dietary behaviors. By presenting standardized information about energy, macronutrients, micronutrients, ingredients, and serving size, food labels help consumers to compare products and select options that better meet their nutritional needs (1, 2). Therefore, food labeling has become an important tool in nutrition policies aimed at preventing obesity and diet-related chronic diseases while supporting healthier food choices worldwide (2, 3).

The potential health benefits of food labeling depend not only on the availability of nutrition information but also on consumers’ ability to understand, interpret, and apply the information during food purchasing decisions. Evidence indicates that regular use of food labels is associated with healthier food choices and improved dietary quality (1, 4, 5). A meta-analysis of 60 intervention studies, including 16 randomized and 44 nonrandomized studies across 11 countries, found that food labeling influenced both consumer dietary behavior and industry practices. Food labeling reduced consumer energy intake by 6.6% and total fat intake by 10.6%, while increasing vegetable consumption by 13.5%. At the industry level, labeling was associated with product reformulation, reducing sodium content by 8.9% and trans-fat content by 64.3% (5). In addition, Christoph et al. (1) found that frequent label users consumed less added sugar and saturated fat, while individuals with higher nutrition literacy were nearly twice as likely to select lower-calorie foods (1). These findings highlight the importance of nutritional knowledge in promoting effective food label use and healthier dietary behaviors.

Moreover, studies conducted in the Middle East have consistently reported relatively low levels of food label use despite widespread recognition of its importance (6, 7). In Saudi Arabia, a cross-sectional study among 572 female university students found that only 27.4% of participants reported always or usually using food labels when purchasing food products, while 38.1% reported using them occasionally. Lack of time and difficulty understanding label terms were identified as major barriers, suggesting the need for educational interventions and clearer, more user-friendly labeling formats (8). Similar findings have been reported in the United Arab Emirates by AlBlooshi et al. (9). Although 89.9% of participants acknowledged the importance of food labeling, only 55.6% reported regularly checking food labels (9). A study from Jordan reported that only 26.4% of consumers were classified as comprehensive food label users, highlighting the need for improved nutrition literacy and supportive public health policies to promote effective food label use (10). Evidence from North Africa also highlights the relevance of nutritional knowledge to dietary practices. In a cross-sectional study of 1,035 Moroccan adults, nutritional knowledge was positively associated with dietary practices, although the study did not specifically assess food label use (11).

Food label use is influenced by a range of individual and contextual factors, including nutritional knowledge, educational level, health literacy, dietary behaviors, age, and socioeconomic characteristics (6, 12). These factors are likely to be interrelated; for example, educational level may influence nutritional knowledge and health literacy and is also closely linked to socioeconomic circumstances. Individuals with greater nutritional knowledge and higher educational level are generally more likely to understand and use food labels when selecting food products, whereas limited nutrition literacy and difficulty interpreting nutrition information may hinder their use (13). In addition, individuals with chronic health conditions or those following a specific diet may rely on nutrition labels to guide food selection and manage their conditions (13). Following a specific diet may reflect broader health-related behaviors, such as greater health consciousness, dietary goals, or the presence of a chronic condition, which may themselves be associated with greater attention to nutrition information. Therefore, these characteristics should be considered as potentially interconnected factors rather than independent determinants of food label use. Understanding these factors is essential for developing interventions that improve consumer’s ability to make informed food choices.

In response to the growing burden of diet-related chronic diseases, Saudi Arabia has strengthened its food labeling regulations as part of broader national strategies to promote healthier dietary behaviors. Following the adoption of the Gulf Standards Organization (GSO) nutrition labeling requirements in 2013, the Saudi Food and Drug Authority (SFDA) mandated nutrition information on packaged foods and, more recently, expanded labeling requirements to food service establishments by introducing calorie, sodium, caffeine, and physical activity equivalent labeling on menus beginning in 2025 (3, 14). Despite these initiatives, food label use remains low, with previous studies identifying insufficient nutritional knowledge and difficulty interpreting label information as common barriers (8).

Evidence regarding the factors associated with food label use among the general Saudi adult population remains limited. Previous studies have focused on specific subgroups, such as university students and women, or on specific regions or cities, thereby limiting the generalizability of their findings (8, 13). Furthermore, few studies have simultaneously examined the associations of nutritional knowledge, educational level, and dietary practices with food label use among Saudi adults. Addressing this gap is important for developing evidence-based educational strategies and strengthening food labeling policies to promote healthier food choices in Saudi Arabia. Therefore, this study aimed to identify factors associated with food label use among Saudi adults. The findings may inform policymakers and public health practitioners in designing targeted interventions to improve nutrition literacy and support healthier food purchasing decisions.

## 2 Methods

### 2.1 Study design, setting and population

A cross-sectional study was conducted among Saudi adults residing in Saudi Arabia using an online survey to assess nutritional knowledge, food label use, and associated factors. Saudi nationals aged 18 years or older were eligible to participate. The structured questionnaire collected information on sociodemographic characteristics, health-related variables, dietary practices, objective and subjective nutritional knowledge, and food label use. Nutrition professionals, defined as participants who had a formal academic or professional background in nutrition, and individuals unable to read Arabic were excluded from the study.

### 2.2 Sample size and sampling methods

Participants were recruited using a non-probability convenience sampling approach through an online questionnaire during January and February 2026. The survey link was initially distributed through the researchers’ social networks and subsequently shared from one participant to another through social media platforms, such as WhatsApp and X, using a snowballing technique. Participation was voluntary, and participants were encouraged to share the survey link with eligible individuals within their social networks. Because the survey link was distributed through social networks and could be shared further, the total number of individuals who received or viewed the invitation could not be determined. Consequently, a survey response rate could not be calculated.

The required sample size was estimated using the standard formula for a single population proportion. Based on an estimated population of 10,520,163 Saudi adults (15), a 95% confidence level (*Z* = 1.96), a 5% margin of error, and an assumed prevalence of 50%, the minimum required sample size was 385 participants. An additional 10% allowance was applied during study planning to account for potentially incomplete questionnaire responses, increasing the target sample size to 423 participants. This allowance was not based on an observed nonresponse rate. The sample-size calculation was intended to establish an initial recruitment target and did not specifically account for the planned multivariable regression analysis.

During the data collection period, 692 responses were received. Following data screening, nine responses were excluded: four participants were non-Saudi and five were younger than 18 years. The final analytic sample included 683 eligible participants.

### 2.3 Questionnaire development

The study questionnaire was adapted from a previously published and validated English-language instrument developed by Al-Barqi et al. (8), with emphasis on the nutritional knowledge and food label use sections. The questionnaire was translated into Arabic using a forward–backward translation process and reviewed by three experts in nutrition and health education to assess its linguistic accuracy, cultural appropriateness and content validity. The item-level content validity index (I-CVI) was 84%. Based on the experts’ feedback, minor revisions were made to improve clarity, simplify wording and enhance readability. The final Arabic version was pre-tested with 20 adults from the target population to assess its clarity and comprehensibility. No further modifications were required before data collection.

### 2.4 Study variables

Covariates were selected *a priori* based on previous literature (8) and their established or plausible associations with food label use (16, 17). These included age, sex, educational level, marital status, monthly income, chronic disease status, following a specific diet, BMI, and objective nutritional knowledge. Covariate selection was not based on statistical significance in univariable analyses.

#### 2.4.1 Sociodemographic characteristics

Participants reported their sex, age, region of residence, educational level, marital status, and monthly household income. Sex was treated as a binary categorical variable (male/female). Age was analyzed as a continuous variable. Marital status was analyzed as a binary categorical variable, categorized as single or ever married (including married, divorced, or widowed). Educational level was coded as an ordinal variable with three categories: high school or below (coded 1), diploma or bachelor’s degree (coded 2), and postgraduate education (coded 3). Monthly household income was coded as an ordinal variable with four categories: ≤5,000 Saudi Riyals (SAR; coded 1), 5,001–15,000 SAR (coded 2), 15,001–25,000 SAR (coded 3), and ≥25,000 SAR (coded 4). In the multivariable regression models, education and income were entered as categorical predictors, with successive-category contrasts comparing each category with the immediately preceding category. The comparison categories are specified in the regression tables.

#### 2.4.2 Health-related variables

Participants reported their chronic disease status, height, and weight. The questionnaire included diabetes, obesity, high cholesterol, cardiovascular disease, hypertension, celiac disease, no chronic disease, and other conditions. For the analysis, chronic disease status was treated as a binary categorical variable; participants reporting at least one chronic condition were classified as having a chronic disease (yes), whereas those reporting no chronic disease were classified as not having a chronic disease (no). Body mass index (BMI) was calculated from self-reported weight and height as weight in kilograms divided by height in meters squared (kg/m^2^). For descriptive reporting, BMI was categorized as underweight (<18.5 kg/m^2^), normal weight (18.5–24.9 kg/m^2^), overweight (25.0–29.9 kg/m^2^), or obese (≥30.0 kg/m^2^), according to World Health Organization criteria (18). For statistical analysis, BMI was treated as a continuous variable.

#### 2.4.3 Dietary practices

Dietary practices were assessed by asking participants whether they were currently following a specific diet. Participants who responded “yes” were further asked about the purpose of the diet, with response options including weight loss, muscle building or increasing muscle mass, improving general health and fitness, managing a specific health condition (e.g., diabetes, hypertension, high cholesterol, or celiac disease), weight gain, or other reasons. For the primary analysis, following a specific diet was treated as a binary categorical variable (yes/no).

#### 2.4.4 Nutritional knowledge

Nutritional knowledge was assessed using both subjective and objective measures. A single item assessed subjective nutritional knowledge, while nine questions assessed objective nutritional knowledge. The nine objective questions covered key dietary concepts, including nutrient composition of foods, healthy food choices, dietary recommendations, and sources of dietary fats and sugars. Correct answers were determined based on established nutrition guidelines and recommendations relevant to each item. Each correct response was assigned one point, while incorrect and “I don’t know” responses received zero points. Scores were summed across all nine items to generate a total objective nutrition knowledge score ranging from 0 to 9, with higher scores indicating higher nutritional knowledge. The internal consistency of the nine objective nutritional knowledge responses was assessed using Cronbach’s alpha in JMP for the full analytical sample (*n* = 683), yielding an *α* of 0.75. Given that the items assess heterogeneous domains of nutritional knowledge, Cronbach’s alpha was interpreted as a descriptive measure of internal consistency rather than evidence validity. No responses were missing for the objective nutritional knowledge items; therefore, no missing-data imputation was required. The nutritional knowledge questions and predefined correct response used to derive the objective nutritional knowledge score are presented in Table 1.

**Table 1:** Scoring of objective nutritional knowledge questions.

No.	Nutrition knowledge question	Response options
1	The amount of calcium in a glass of whole milk compared to a glass of skimmed milk is?	Higher
		Lower
		Approximately the same ✓
		I don’t know
2	One healthy way to add flavor to food without adding extra fat or salt is to add:	Chicken stock cubes
		Fresh or dried herbs and spices ✓
		Cooking cream
		I don’t know
3	Which one of the following nutrients has the most calories for the same weight of food?	Protein
		Fat ✓
		Carbohydrate
		I don’t know
4	Which of these types of fats should you eat less to maintain your health?	Trans fat ✓
		Saturated fat
		Omega-3 fat
		I don’t know
5	How many servings of fruit and vegetables do you need per day to eat as a minimum?	1–2 servings
		3–4 servings
		5 or more servings ✓
		I don’t know
6	Which of the following items are typically high in added sugar?	Plain yogurt
		Ketchup ✓
		Diet energy drinks
		I don’t know
7	How many times should you eat oily fish (e.g., salmon and tuna) per week?	Daily
		Once/week
		2–3 times/week ✓
		I don’t know
8	Which of the following foods count as starchy foods?	Cheese
		Potatoes ✓
		Nuts
		I don’t know
9	Which of these foods has the most trans-fat?	Biscuits ✓
		Olive oil
		Eggs
		I don’t know

Nutritional knowledge items and predefined correct responses were adapted from Al-Barqi et al. (8), with nutritional information supported, where applicable, by relevant World Health Organization (WHO) dietary recommendations (24). ✓ indicates the predefined correct response.

Subjective nutritional knowledge was evaluated through a self-assessment question in which participants were asked to rate their knowledge of nutrition on a four-point scale: no knowledge at all, low knowledge, moderate knowledge, or excellent knowledge. Responses were coded from 0 to 3, with higher scores indicating greater subjective nutritional knowledge. This measure was included to capture participants’ self-perceived understanding of nutrition and healthy eating and was used in the sensitivity analyses.

#### 2.4.5 Food label use

Food label use was assessed using nine items evaluating how frequently participants used food label information during food purchasing decisions. The items were adapted from the Food and Drug Administration (FDA) Health and Diet Survey (19) and Williams (20), as reported in the original instrument by Al-Barqi et al. (8). The nine items assessed: (1) reading food labels when purchasing food products; using food label information to (2) select brands, (3) compare food products, (4) verify marketing claims, (5) check fat content, (6) check calorie content, (7) assist with meal planning, and (8) determine serving size; and (9) the extent to which food purchases were not influenced by information provided on food labels. Responses were measured on a five-point frequency scale ranging from “never” to “always.” Items 1–8 were scored from 1 (never) to 5 (always), whereas the negatively worded ninth item was reverse-scored from 5 (never) to 1 (always), ensuring that higher scores consistently represented greater food label use. The nine items were summed to generate a composite score ranging from 9 to 45, with higher scores indicating greater engagement with and use of food label information. The items were combined because they capture related behavioral aspects of food label use, including accessing, evaluating, and applying label information to food-related decisions. The internal consistency of the nine-item scale was assessed using Cronbach’s alpha in the full analytical sample (*n* = 683), yielding *α* = 0.89. No responses were missing for the food label use items; therefore, no missing-data imputation was required.

### 2.5 Statistical analysis

Data were cleaned and analyzed using JMP Statistical Software, Version 19 (SAS Institute Inc., Cary, NC, USA).

Descriptive statistics were used to summarize participant characteristics. Continuous variables were presented as means and standard deviations (SD), whereas categorical variables were presented as frequencies and percentages.

To identify factors associated with food label use, multivariable linear regression analysis was performed with the food label use score as the dependent variable. Independent variables included objective nutritional knowledge score, age, sex, educational level, marital status, monthly income, chronic disease status, following a specific diet, and BMI. Model assumptions were assessed using diagnostic plots and statistics. Linearity was evaluated by examining residual plots, normality of residuals using a normal quantile (Q–Q) plot, and homoscedasticity by examining residuals versus predicted values. Potential influential observations and outliers were assessed using residual and influence diagnostics. Multicollinearity among predictors was evaluated using variance inflation factors (VIFs).

A sensitivity analysis was conducted to examine whether the main associations observed in the primary model were also present when nutritional knowledge was assessed using a complementary measure. Subjective nutritional knowledge replaced objective nutritional knowledge in the multivariable linear regression model, while all other model variables remained unchanged. The results were compared with those of the primary analysis to determine whether the observed associations were consistent when nutritional knowledge was assessed using a subjective rather than an objective measure.

Regression coefficients (*B*), 95% confidence intervals (CI), and *p*-values were reported. Statistical significance was defined as *p* < 0.05.

## 3 Results

### 3.1 Descriptive statistics

A total of 683 participants were included in the final analysis. Table 2 presents the sociodemographic and health-related characteristics of the study participants.

**Table 2:** Sociodemographic and health-related characteristics of the study participants (*N* = 683).

Sociodemographic data	No.	(%)
Sex		
Male	212	31.0
Female	471	69.0
Age (years), Mean ± SD	30.8 ± 11.9	
Region of residence		
Central region	348	51.0
Western region	120	17.5
Eastern region	105	15.4
Southern region	57	8.3
Northern region	53	7.8
Education level		
High school or below	108	15.8
Diploma or bachelor’s degree	509	74.5
Postgraduate education	66	9.7
Marital status		
Single	392	57.4
Ever married	291	42.6
Monthly income (SAR)		
≤5,000	350	51.2
5,001–15,000	198	29.0
15,001–25,000	87	12.7
≥25,000	48	7.1
Chronic disease status		
No	545	79.8
Yes	138	20.2
BMI (kg/m^2^)		
Underweight	60	8.8
Normal weight	250	36.6
Overweight	240	35.1
Obese	133	19.5
Following a specific diet		
No	414	60.6
Yes	269	39.4

Females represented the majority of the sample (69.0%), while males accounted for 31.0%. The mean age of participants was 30.8 years (SD = 11.9). Approximately half of the participants resided in the Central Region of Saudi Arabia (51.0%), followed by the Western Region (17.5%) and Eastern Region (15.4%). The Southern and Northern Regions represented 8.3 and 7.8% of the sample, respectively. Most participants had a diploma or bachelor’s degree (74.5%), while 15.8% had a high school education or below and 9.7% had postgraduate education. Regarding marital status, 57.4% of participants were single, while 42.6% were ever married. In terms of income, approximately half of the participants (51.2%) reported a monthly income of SAR 5,000 or less, whereas only 7.1% reported a monthly income of SAR 25,000 or more.

Regarding health-related characteristics, 20.2% of participants reported having a chronic disease. In terms of body weight status, 36.6% of participants were classified as having normal weight, 35.1% as overweight, 19.5% as obese, and 8.8% as underweight. Overall, 54.6% of participants were classified as overweight or obese. Additionally, 39.4% of participants reported following a specific diet at the time of the survey.

Table 3 presents the distribution of subjective nutritional knowledge, objective nutritional knowledge, food label use, and BMI among the study participants.

**Table 3:** Distribution of nutritional knowledge, food label use, and BMI (*N* = 683).

Variable	Range	Mean ± SD
Subjective nutritional knowledge	0–3	1.72 ± 0.80
Objective nutritional knowledge	0–9	4.34 ± 1.90
Food label use score	9–45	27.51 ± 7.37
BMI (kg/m^2^)	15–69	25.59 ± 5.86

The mean subjective nutritional knowledge score was 1.72 (SD = 0.80), while the mean objective nutritional knowledge score was 4.34 (SD = 1.90). The mean food label use score was 27.51 (SD = 7.37), and the mean BMI was 25.59 kg/m^2^ (SD = 5.86).

Table 4 presents the distribution of responses to the nine objective nutritional knowledge items among 683 participants. The highest proportions of correct responses were observed for identifying starchy foods (80.2%), selecting a healthier way to add flavor to food (79.4%), and identifying foods that may contain trans fats (71.4%). In contrast, the lowest proportion of correct responses was observed for the recommended daily intake of fruits and vegetables (7.2%), followed by the calcium content of whole versus skimmed milk (29.6%). Correct-response rates for the remaining items ranged from 42.6 to 55.5%.

**Table 4:** Responses to objective nutritional knowledge questions among Saudi adults (*n* = 683).

Objective nutritional knowledge item	Correct, *n* (%)	Incorrect or do not know, *n* (%)
1. The amount of calcium in a glass of whole milk compared to a glass of skimmed milk is?	202 (29.6)	481 (70.4)
2. One healthy way to add flavor to food without adding extra fat or salt is to add:	542 (79.4)	141 (20.6)
3. Which one of the following nutrients has the most calories for the same weight of food?	303 (44.4)	380 (55.6)
4. Which of these types of fats should you eat less to maintain your health?	379 (55.5)	304 (44.5)
5. How many servings of fruit and vegetables do you need per day to eat as a minimum?	49 (7.2)	634 (92.8)
6. Which of the following items are typically high in added sugar?	292 (42.8)	391 (57.2)
7. How many times should you eat oily fish (e.g., salmon and tuna) per week?	291 (42.6)	392 (57.4)
8. Which of the following foods count as starchy foods?	548 (80.2)	135 (19.8)
9. Which of these foods has the most trans-fat?	488 (71.4)	195 (28.6)

Correct responses were scored as 1; incorrect and “Do not know” responses were scored as 0.

### 3.2 Factors associated with food label use (primary analysis)

Table 5 presents the multivariable linear regression analysis examining factors associated with food label use among 683 participants.

**Table 5:** Multivariable linear regression analysis of factors associated with food label use among Saudi adults (*N* = 683).

Predictor	*B*	SE	*t*	95% CI	*p*-value
Objective nutritional knowledge score	0.715	0.25	2.84	0.220, 1.209	0.004
Age (years)	−0.065	0.03	−1.67	−0.142, 0.011	0.095
Male (vs. female)	−0.404	0.30	−1.34	−0.995, 0.187	0.180
Education					
Diploma/bachelor’s degree vs. high school or below	1.677	0.76	2.20	0.182, 3.172	0.028
Postgraduate vs. diploma/bachelor’s degree	1.345	1.04	1.29	−0.709, 3.398	0.199
Monthly income					
SAR 5,001–15,000 vs. ≤ SAR 5,000	−0.454	0.69	−0.65	−1.827, 0.919	0.516
SAR 15,001–25,000 vs. SAR 5,001–15,000	0.951	0.92	1.02	−0.871, 2.772	0.306
≥SAR 25,000 vs. SAR 15,001–25,000	−0.332	1.28	−0.26	−2.847, 2.183	0.796
Single (vs. ever married)	−0.654	0.43	−1.49	−1.517, 0.210	0.138
No chronic disease (vs. chronic disease)	0.653	0.43	1.73	−0.088, 1.394	0.084
Not following a specific diet (vs. following a specific diet)	−2.324	0.27	−8.43	−2.866, −1.783	<0.001
BMI (kg/m^2^)	0.032	0.05	0.60	−0.074, 0.138	0.549

Model fit: *n* = 683; *R*^2^ = 0.129; adjusted *R*^2^ = 0.113; *F*(12, 670) = 8.26, *p* < 0.001.

*B*, unstandardized regression coefficient; CI, confidence interval; BMI, body mass index; SAR, Saudi Riyal.

Objective nutritional knowledge was positively associated with food label use (*B* = 0.715, 95% CI: 0.220–1.209, *p* = 0.004), indicating greater use of food labels among participants with higher knowledge.

Educational level was also associated with food label use. Participants with a diploma or bachelor’s degree had significantly higher food label use scores than those with a high school education or lower (*B* = 1.677, 95% CI: 0.182–3.172, *p* = 0.028). However, no additional benefit was observed among participants with postgraduate education (*p* = 0.199).

Participants not following a specific diet had significantly lower food label use scores than those following a specific diet (*B* = −2.324, 95% CI: −2.866 to −1.783, *p* < 0.001), representing the largest regression coefficient among the factors examined.

Age, sex, marital status, monthly income, BMI, and chronic disease status were not significantly associated with food label use (all *p* > 0.05).

Regression diagnostics indicated no substantial violations of linearity, normality of residuals, or homoscedasticity, and no influential observations materially affected the model estimates. VIF values ranged from 1.01 to 1.93, indicating no evidence of problematic multicollinearity among the predictors.

### 3.3 Factors associated with food label use (sensitivity analysis)

To examine whether the associations observed in the primary model were also present when nutritional knowledge was assessed using a complementary measure, a sensitivity analysis was conducted by replacing objective nutritional knowledge with subjective nutritional knowledge in the multivariable model (Table 6). The sensitivity model included 683 participants.

**Table 6:** Sensitivity analysis: multivariable linear regression examining factors associated with food label use among Saudi adults (*N* = 683).

Predictor	*B*	SE	*t*	95% CI	*p*-value
Subjective nutritional knowledge score	2.440	0.33	7.33	1.786, 3.094	<0.001
Age (years)	−0.076	0.03	−2.00	−0.150, −0.001	0.046
Male (vs. female)	−0.409	0.03	−1.40	−0.981, 0.163	0.161
Education					
Diploma/bachelor’s degree vs. high school or below	1.537	0.29	2.09	0.090, 2.984	0.037
Postgraduate vs. Diploma/Bachelor’s degree	1.275	0.73	1.26	−0.712, 3.261	0.208
Single (vs. married)	−0.721	0.42	−1.69	−1.556, 0.114	0.091
Monthly income					
SAR 5,001–15,000 vs. ≤ SAR 5,000	−0.435	0.67	−0.64	−1.764, 0.893	0.520
SAR 15,001–25,000 vs. SAR 5,001–15,000	0.956	0.89	1.07	−0.805, 2.716	0.287
≥SAR 25,000 vs. SAR 15,001–25,000	−0.574	1.23	−0.46	−3.004, 1.857	0.643
No chronic disease (vs. chronic disease)	0.778	0.36	2.13	0.060, 1.496	0.034
Not following a specific diet (vs. following a specific diet)	−1.901	0.27	−6.97	−2.436, −1.366	<0.001
BMI (kg/m^2^)	0.008	0.05	0.16	−0.095, 0.111	0.874

Model fit: *R*^2^ = 0.184; adjusted *R*^2^ = 0.169; *F*(12, 670) = 12.58, *p* < 0.001; *n* = 683.

*B*, unstandardized regression coefficient; CI, confidence interval; BMI, body mass index; SAR, Saudi Riyal.

Subjective nutritional knowledge was positively associated with food label use (*B* = 2.440, 95% CI: 1.786–3.094, *p* < 0.001), indicating that participants who perceived themselves as having greater nutritional knowledge had higher food label use scores.

Consistent with the primary analysis, participants with a diploma or bachelor’s degree had higher food label use scores than those with a high school education or below (*B* = 1.537, 95% CI: 0.090–2.984, *p* = 0.037). Similarly, participants not following a specific diet had lower food label use scores than those following a specific diet (*B* = −1.901, 95% CI: −2.436 to −1.366, *p* < 0.001). Age, sex, marital status, monthly income, postgraduate education, and BMI were not significantly associated with food label use.

Age showed a small negative association with food label use in the sensitivity model (*B* = −0.076, 95% CI: −0.150 to −0.001, *p* = 0.046). Participants without a chronic disease also had higher food label use scores than those with a chronic disease (*B* = 0.778, 95% CI: 0.060 to 1.496, *p* = 0.034); however, these associations were not statistically significant in the primary model and should therefore be interpreted cautiously. Sex, marital status, monthly income, postgraduate education, and BMI were not significantly associated with food label use in the sensitivity model. Overall, objective and subjective nutritional knowledge provided complementary evidence of a positive association between nutritional knowledge and food label use. The associations involving diploma/bachelor’s education and following a specific diet were also observed in both models. However, the differences in the age and chronic disease findings indicate that not all associations were consistent across the two models.

## 4 Discussion

This study examined factors associated with food label use among Saudi adults using data from a large cross-sectional survey that included participants from multiple regions of Saudi Arabia. Three main findings were identified. First, higher objective nutritional knowledge was independently associated with greater food label use. Second, higher educational level was positively associated with food label use, particularly among participants with a diploma or bachelor’s degree compared with those with a high school education or below. Third, not following a specific diet was significantly associated with lower food label use. These associations remained consistent in the sensitivity analysis, in which subjective nutritional knowledge replaced objective nutritional knowledge.

The findings of this study indicate that each one-unit increase in the nutritional knowledge score was associated with a 0.72-unit increase in the food label use score. In comparison, participants with a diploma/bachelor’s degree had food label use scores 1.68 points higher than those with high school education or below, while participants not following a specific diet had scores 2.32 points lower than those following a specific diet. These findings indicate that although nutritional knowledge was significantly associated with food label use, the estimated difference associated with a one-point increase in knowledge was smaller than the differences observed across the significant education and dietary-practice categories This finding is consistent with previous evidence from Saudi Arabia and internationally showing a positive association between nutritional knowledge and food label use (8, 21, 22). The questionnaire used in the present study was adapted from Al-Barqi et al. (8), providing some comparability in the measurement approach. However, Al-Barqi et al. (8) studied female health college students from a single university, whereas the present study included Saudi adults from multiple regions and used a composite food label use score as the outcome. Therefore, direct comparison of the magnitude of associations should be made cautiously. Similarly, studies included in the systematic review by Campos et al. (21) varied in their populations, definitions, measurement instruments, and scoring approaches. Such methodological differences may partly explain variation in the magnitude and consistency of associations reported across studies. Overall, the available evidence supports an association between nutritional knowledge and food label use, while highlighting the importance of considering differences in measurement and study populations when interpreting findings across studies. However, given the cross-sectional design, the direction of this association cannot be established. Although greater nutritional knowledge may facilitate the use and interpretation of food labels, frequent label use may also contribute to greater nutritional knowledge through repeated exposure to nutrition information. Therefore, interventions should not assume that improving nutritional knowledge alone will necessarily increase food label use. Rather, interventions could integrate nutrition education with practical strategies that support consumers in locating, interpreting, and applying label information during food selection.

Educational level was also positively associated with food label use. Participants with a diploma or bachelor’s degree had higher food label use scores than those with a high school education or below. This finding is consistent with previous Saudi and international studies (8, 21). One possible explanation is that educational level may be related to greater ability to locate, interpret, and apply nutrition information.

Participants who were not following a specific diet had food label use scores that were 2.32 points lower than those of participants following a specific diet, after adjustment for other factors. This difference was larger in magnitude than the association observed for a one-point increase in objective nutritional knowledge, suggesting that dietary practices may have a more substantial association with food label use. Similar findings have been reported previously (1, 11, 21). In the present study, reported reasons for following a specific diet included weight loss, muscle building, improving general health and fitness, managing a health condition, weight gain, and other purposes. Therefore, the observed association may reflect differences in dietary goals, health consciousness, or attention to nutritional information rather than the effects of a particular type of diet or a medically prescribed dietary regimen. The direction of this association cannot be determined from the cross-sectional design. Following a specific diet may be associated with greater attention to nutrition information; conversely, individuals who frequently consult food labels may be more likely to adopt or maintain specific dietary practices. Longitudinal studies are needed to clarify the temporal relationship between dietary practices and food label use.

In the primary analysis, age, sex, marital status, monthly income, BMI, and chronic disease status were not independently associated with food label use. These findings differ from some previous studies, including the systematic review by Campos et al. (21), which documented variation in food label use across demographic groups. However, previous research suggests that sociodemographic characteristics may have weaker associations with food label use when health-related and motivational factors are considered simultaneously (23). One possible explanation is that adjustment for nutritional knowledge, educational level, and dietary practices in the present study may have weakened the associations between sociodemographic characteristics and food label use.

The sensitivity analysis showed that the associations of educational level and dietary practices with food label use remained consistent when subjective nutritional knowledge replaced objective nutritional knowledge, supporting the robustness of these findings. However, age and chronic disease status was significantly associated with food label use in the sensitivity analysis, whereas no significant association was observed in the primary analysis. Age showed a small negative association with food label use in the sensitivity model using subjective nutritional knowledge (*B* = −0.076, 95% CI: −0.150 to −0.001, *p* = 0.046), corresponding to an estimated 0.076-point lower food label use score per additional year of age. Although this association reached statistical significance, its magnitude was small relative to the 9–45-point food label use scale, suggesting limited practical significance. Moreover, age was not significantly associated with food label use in the primary model using objective nutritional knowledge. The age-related finding should therefore be interpreted cautiously. Participants without a chronic disease had slightly higher food label use scores than those with a chronic disease in the sensitivity model using subjective nutritional knowledge (*B* = 0.778, 95% CI: 0.060–1.496, *p* = 0.034), whereas this association was not significant in the primary model. This difference may reflect the distinct constructs captured by objective and subjective nutritional knowledge, which may alter the estimated association between chronic disease and food label use. As the association was significant only in the sensitivity model, this finding should be interpreted cautiously and requires further investigation.

This study has several strengths. It included a relatively large sample of Saudi adults from multiple regions and examined both objective and subjective measures of nutritional knowledge using multivariable analyses. The study also included a range of sociodemographic, dietary, and health-related characteristics, allowing the independent associations of nutritional knowledge and other factors with food label use to be examined. In addition, the study addresses an important gap in the Saudi literature, where previous research has largely focused on selected populations, such as university students or women, or has been limited to individual regions (8, 13).

Despite these strengths, several limitations should be acknowledged. First, the cross-sectional design precludes causal inferences and does not establish the temporal direction of the observed associations. Second, participants were recruited using non-probability convenience and snowball sampling through an online, self-administered questionnaire, which may have introduced selection and self-report biases. Online recruitment may have preferentially attracted individuals with greater internet access, higher educational level, or greater interest in health and nutrition, potentially resulting in an overestimation of food label use. Furthermore, because the sampling approach did not provide all eligible Saudi adults with a known probability of selection, the sample-size calculation and its assumed 5% margin of error do not establish the statistical precision or representativeness of the final sample. Third, although participants were recruited from multiple regions, females (69.0%) and participants from the Central Region (51.0%) were disproportionately represented. These sampling characteristics may limit the generalizability of the findings to the broader Saudi adult population. Future studies should use more geographically and gender-balanced sampling strategies to improve representativeness.

This study has important implications for public health practice. The present findings suggest that food labeling policies could be complemented by interventions aimed at improving nutrition literacy and consumers’ ability to use nutrition information effectively. Such interventions could include nutrition-label literacy education, practical guidance on interpreting key nutrition information, and targeted educational campaigns to improve consumers’ ability to use food labels effectively. These approaches may be particularly relevant for individuals with lower nutritional knowledge or educational levels and for those who are not currently following a specific diet.

In conclusion, nutritional knowledge, educational level, and following a specific diet were independently associated with food label use among Saudi adults. The consistency of the main associations when subjective nutritional knowledge replaced objective nutritional knowledge supports the main findings. These findings suggest that strengthening nutrition literacy may enhance consumers’ use of food labels and potentially increase the effectiveness of food labeling policies. Longitudinal and intervention studies are needed to determine whether improvements in nutritional knowledge and nutrition literacy lead to sustained changes in food label use and dietary behavior.

## Data Availability

The raw data supporting the conclusions of this article will be made available by the authors, without undue reservation.
